# Analysis and Prediction of the Metabolic Stability of Proteins Based on Their Sequential Features, Subcellular Locations and Interaction Networks

**DOI:** 10.1371/journal.pone.0010972

**Published:** 2010-06-04

**Authors:** Tao Huang, Xiao-He Shi, Ping Wang, Zhisong He, Kai-Yan Feng, LeLe Hu, Xiangyin Kong, Yi-Xue Li, Yu-Dong Cai, Kuo-Chen Chou

**Affiliations:** 1 Key Laboratory of Systems Biology, Shanghai Institutes for Biological Sciences, Chinese Academy of Sciences, Shanghai, People's Republic of China; 2 Shanghai Center for Bioinformation Technology, Shanghai, People's Republic of China; 3 Institute of Systems Biology, Shanghai University, Shanghai, People's Republic of China; 4 Institute of Health Sciences, Shanghai Institutes for Biological Sciences, Chinese Academy of Sciences and Shanghai Jiao Tong University School of Medicine, Shanghai, People's Republic of China; 5 CAS-MPG Partner Institute for Computational Biology, Shanghai Institutes for Biological Sciences, Chinese Academy of Sciences, Shanghai, People's Republic of China; 6 State Key Laboratory of Medical Genomics, Ruijin Hospital, Shanghai Jiaotong University, Shanghai, People's Republic of China; 7 Centre for Computational Systems Biology, Fudan University, Shanghai, People's Republic of China; 8 Gordon Life Science Institute, San Diego, California, United States of America; Griffith University, Australia

## Abstract

The metabolic stability is a very important idiosyncracy of proteins that is related to their global flexibility, intramolecular fluctuations, various internal dynamic processes, as well as many marvelous biological functions. Determination of protein's metabolic stability would provide us with useful information for in-depth understanding of the dynamic action mechanisms of proteins. Although several experimental methods have been developed to measure protein's metabolic stability, they are time-consuming and more expensive. Reported in this paper is a computational method, which is featured by (1) integrating various properties of proteins, such as biochemical and physicochemical properties, subcellular locations, network properties and protein complex property, (2) using the mRMR (Maximum Relevance & Minimum Redundancy) principle and the IFS (Incremental Feature Selection) procedure to optimize the prediction engine, and (3) being able to identify proteins among the four types: “short”, “medium”, “long”, and “extra-long” half-life spans. It was revealed through our analysis that the following seven characters played major roles in determining the stability of proteins: (1) KEGG enrichment scores of the protein and its neighbors in network, (2) subcellular locations, (3) polarity, (4) amino acids composition, (5) hydrophobicity, (6) secondary structure propensity, and (7) the number of protein complexes the protein involved. It was observed that there was an intriguing correlation between the predicted metabolic stability of some proteins and the real half-life of the drugs designed to target them. These findings might provide useful insights for designing protein-stability-relevant drugs. The computational method can also be used as a large-scale tool for annotating the metabolic stability for the avalanche of protein sequences generated in the post-genomic age.

## Introduction

Proteins are inherently dynamic molecules of marginal stability. Many marvelous biological functions of proteins are realized through their internal motions [1], [2], [3], [4]. The physicochemical stability and flexibility are balanced with each other. They are also thought as intimately correlated with their intramolecular fluctuations and various other dynamic processes [5]. Protein flexibility facilitates adaptation and recognition [6] in diverse molecular events, such as switch between active and inactive states [7], allosteric transition [8], intercalation of drugs into DNA [9], cooperative effects [10], and assembly of microtubules [11]. It is also essential for in-depth understanding the M2 proton channel gating and inhibition mechanism [3], [12], [13], [14], the switch mechanism of human Rab5a [15], the inhibition mechanism of PTP1B [16], the metabolic mechanism [17], and the action mechanism of calmodulin [18], [19]. These properties present unique challenges to the pharmaceutical scientists attempting to develop protein-stability-relevant drugs [20], [21], [22].

Traditional methods of measuring protein's metabolic stability rely on either pulse-chase metabolic labeling or administration of protein synthesis inhibitors followed by half-life biochemical analysis of the abundance of the protein concerned at multiple time points during the chase period. Highly regulated proteins tend to be present in low amounts. Since even mass spectrometry plus failed to detect low-abundance proteins, study about protein's metabolic stability remains far from complete yet although it is of critical importance for drug development. Recently, it was reported that high-throughput systematic approaches for the analysis of global metabolic stability were taken by using a fluorescence-based system to monitor metabolic stability at the single-cell level [23]. In this regard, however, computational approaches would be much more efficient not only in timely providing the information about the stability of query proteins but also in helping analyze what factors play major roles to the metabolic stability. This study was initiated in an attempt to develop a computational method for investigating the metabolic stability of proteins in terms of their biochemical and physicochemical properties or features. Our results suggest that KEGG enrichment scores, subcellular locations, polarity, amino acids composition, hydrophobicity, secondary structure propensity, and number of protein complexes, play irreplaceable roles for protein's metabolic stability. Moreover, we predicted the metabolic stability of drug target proteins using the selected features and found an intriguing correlation between the predicted metabolic stability of some proteins and the real half-life of the drugs designed to target them.

## Materials and Methods

### Data set

As elucidated in a recent review [24], to develop an effective statistical method for predicting protein attributes, one of the indispensable things is a valid benchmark dataset. Here, protein stability data were taken from Yen's work [23]. We downloaded ORFs from hORFeome v5.1 library (http://horfdb.dfci.harvard.edu/), and translated ORFs to protein sequences using transeq in Emboss [25]. Proteins with the length shorter than 50 and longer than 2700, were excluded. In Yen's work, protein samples were divided into four groups according to their PSI (protein stability index): (1) short half-life (PSI<2), (2) medium half-life (2≤PSI<3), (3) long half-life (3≤PSI<4), and (4) extra-long half-life (PSI≥4). After being thus processed, our dataset consist of 223 short half-life proteins, 446 medium half-life proteins, 706 long half-life proteins and 496 extra-long half-life proteins. For reader's convenience, these sequences (classified into above four groups) are given in Dataset S1.

### Biochemical and physicochemical description of proteins

In order to formulate protein samples of different sequence lengths with vectors of a uniform dimension, let us adopt the concept of pseudo amino acid composition (PseAAC) [24], [26], [27]. The concrete procedures are that the biochemical and physicochemical properties [28], [29], [30], [31] are singled out from a protein sequence according to the following seven aspects: (1) amino acid composition (AAC) [32], (2) secondary structure propensity, (3) hydrophobicity, (4) polarizability, (5) solvent accessibility, (6) normalized van der Waals volume, and (7) polarity [33].

Of the above seven types of properties, except for AAC (the occurrence frequencies of the 20 native amino acids for a given protein [34]) that is an extensive quantity reflecting the global or overall feature of a protein, all the other six types are associated with a single amino acid in a given protein sequence position and hence belong to a localized quantity.

The six local types of properties can each be classified into two or three categories. For example, for the secondary structure propensity, each amino acid can be classified as: helix, strand or coil, as predicted by SSpro [35]. For solvent accessibility: buried or exposed to solvent, as predicted by ACCpro [36]. For the other four types of properties, i.e., hydrophobicity, polarizability, normalized van der Waals volume and polarity, each of the constituent amino acids can also be classified into three categories in a similar way according to their values. In terms of hydrophobicity, there are three groups of amino acid: polar (R, K, E, D, Q, N), neutral (G, A, S, T, P, H, Y) and hydrophobic (C, V, L, I, M, F, W) [37]. In terms of polarizability, there are three groups of amino acid: 0–0.108 (G, A, S, D, T), 0.128–0.186 (C, P, N, V, E, Q, I, L) and 0.219–0.409 (K, M, H, F, R, Y, W) [38]. In terms of normalized van der Waals volume, there are three groups of amino acid: 0–2.78 (G, A, S, C, T, P, D), 2.95–4.0 (N, V, E, Q, I, L) and 4.43–8.08 (M, H, K, F, R, Y, W) [38]. In terms of polarity, there are three groups of amino acid: 4.9–6.2 (L, I, F, W, C, M, V, Y), 8.0–9.2 (P, A, T, G, S) and 10.4–13.0 (H, Q, R, K, N, E, D) [39].

Now, the problem is how to generate the corresponding global quantity by integrating the localized quantities over an entire protein sequence. To realize this, let us consider the hydrophobicity first. In this study, the hydrophobicity of an amino acid is classified as: P (polar), N (neutral), or H (hydrophobic). Thus, for a protein sequence, say, “MSDKPDMAEIEKFSKETIEQEKQAGESTQEKNPLPMLLPATDKSKLKKTE”, it can be coded as “HNPPNPHNPHPPHNPPNHPPPPPNNPNNPPPPNHNHHHNNNPPNPHPPNP”.

For the above coded sequence, the following three extensive quantities can be derived: 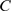 (composition), 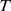 (transition), and 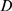 (distribution). 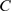 refers to the global percent composition of each of the three groups (i.e., P, N, and H) in the coded sequence; 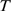 to the percent frequencies with which the code letter changes to another along the entire length of the coded sequence; and 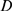 to the distribution pattern of the code letters along the sequence, measuring the percentage of the sequence length within which the first, 25%, 50%, 75%, and 100% of each of the three code letters is located.

**Figure 1:**
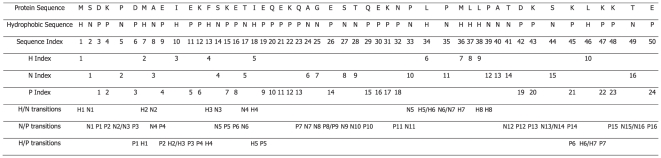
How to compute the 21 hydrophobic feature components from protein sequence. According to the hydrophobicity of each amino acid, the protein sequence “MSDKPDMAEIEKFSKETIEQEKQAGESTQEKNPLPMLLPATDKSKLKKTE” was converted to a hydrophobic sequence “HNPPNPHNPHPPHNPPNHPPPPPNNPNNPPPPNHNHHHNNNPPNPHPPNP”. It is composed of 10 Hs, 16 Ns and 24 Ps. There are totally 31 transitions in the sequence, with 8 between H and N, 16 between N and P, and 7 between H and P. Based on the composition, transition, and distribution of H, N, P, 21 hydrophobic feature components of this protein can be calculated.

Take the above coded sequence of 50 letters as an example. It is composed of 10 Hs, 16 Ns and 24 Ps, as shown in **Figure 1**. Thus, we have the composition 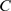 = (10/50 = 20.0%, 16/50 = 32%, 24/50 = 48%) for H, N and P respectively. For the transition feature 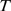, there are totally 31 transitions in the sequence, with 8 between H and N, 16 between N and P, and 7 between H and P, so that we have 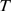 = (8/31 = 25.81%, 16/31 = 51.61% and 7/31 = 22.58%). As for the distribution 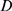, the first, 25%, 50%, 75% and 100% of H are located at the positions of 1st, 10th, 18th, 37th, and 46th in the coded sequence, respectively. Thus, the distribution 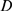 for H is 1/50 = 2%, 10/50 = 20%, 18/50 = 36%, 37/50 = 74%, and 46/50 = 92%. Likewise, the distribution 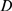 for N is 4%, 28%, 54%, 78%, and 98%; and that for P is 6%, 24%, 44%, 64%, and 100%. Accordingly, we have 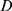 = (2%, 20%, 36%, 74%, 92%, 4%, 28%, 54%, 78%, 98%, 6%, 24%, 44%, 64%, and 100%). Combining 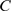, 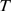 and 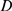, we have a total of 21 elements.

For the “secondary structure”, “polarizability”, “normalized van der Waals volume” and “polarity”, each of them is also classified into three categories and hence would also generate **21** elements in a similarly way as described above for the case of “hydrophobicity”.

For the “solvent accessibility”, since it is classified into two categories, the combination of 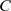, 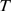 and 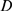 for the sequence coded according to the “solvent accessibility” would only generate 7 elements rather than 21.

Now for the “AAC” we have 20 elements [34]; for the “solvent accessibility”, 7 elements; and for each of all the other five types of protein properties, 21 elements. Combining all these extensive quantities together, we have an augmented extensive quantity containing (5×21+20+7) = 132 elements, as listed in **Table 1** for the details. Furthermore, some more elements should also be included as will be illustrated below.

**Table 1:** The 132 biochemical and physicochemical feature components of proteins.

Biochemical and physicochemical description	Vector index	Amino acid category	Protein Feature category	Protein Feature description	Number of vector components	Index of vector components	Total Number
Hydrophobicity	V1:V21	P: r|k|e|d|q|n	*C* (composition)	Composition of P, N, H	3	V1:V3	21
		N: g|a|s|t|p|h|y	*T* (transition)	Transition of PN, PH, NH	3	V4:V6	
		H: c|v|l|i|m|f|w	*D* (distribution)	Distribution of P, N, H	15	V7:V21	
Secondary structure	V22:V42	SSpro	*C* (composition)	Composition of H, E, C	3	V22:V24	21
		H: helix	*T* (transition)	Transition of HE, HC, EC	3	V25:V27	
		E: strand	*D* (distribution)	Distribution of H, E, C	15	V28:V42	
		C:coil					
Solvent accessibility	V43:V49	ACCpro	*C* (composition)	Composition of H	1	V43	7
		H: hidden	*T* (transition)	Transition of HE	1	V44	
		E: exposed	*D* (distribution)	Distribution of H	5	V45:V49	
Normalized van der Waals volume	V50:V70	H: g|a|s|c|t|d|p	*C* (composition)	Composition of H, E, C	3	V50:V52	21
		E: n|v|e|q|i|l	*T* (transition)	Transition of HE, HC, EC	3	V53:V55	
		C:m|h|k|f|r|y|w	*D* (distribution)	Distribution of H, E, C	15	V56:V70	
Polarity	V71:V91	H: l|i|f|w|c|m|v|y	*C* (composition)	Composition of H, E, C	3	V71:V73	21
		E: g|a|t|p|s	*T* (transition)	Transition of HE, HC, EC	3	V74:V76	
		C: h|q|r|k|n|e|d	*D* (distribution)	Distribution of H, E, C	15	V77:V91	
Polarizability	V92:V112	H: g|a|s|d|t	*C* (composition)	Composition of H, E, C	3	V92:V94	21
		E: c|p|n|v|e|q|i|l	*T* (transition)	Transition of HE, HC, EC	3	V95:V97	
		C: k|m|h|f|r|y|w	*D* (distribution)	Distribution of H, E, C	15	V98:V112	
Amino Acids Composition	V113:V132	r, k, e, d, q, n, g, a, s, t, p, h, y, c, v, l, i, m, f, w	*C* (composition)	20	20		

### Subcellular location description of proteins

The function of a protein is closely correlated with its subcellular location [40], [41]. In view of this, the prediction power would be improved by incorporating the protein subcellular location information. Unfortunately, only a small amount of proteins have subcellular locations annotated in UniProt [42]. To make up this, the subcellular locations for most proteins were predicted based on the sequence similarity evaluated by BLAST [43]. If the BLAST score of a query protein with a location-known protein was greater than 120, they were considered similar with the query protein. The subcellular locations of the query protein were the intersection of subcellular locations of its sequence similar location-known proteins. Since there were 22 subcellular locations, the subcellular location features of each protein can be represented by a 22-dimensional vector, namely 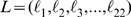, where 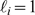 if the protein is located at the 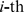 subcellular location site; otherwise, 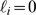. It is instructive to point out that one can also use the web-server predictor Euk-mPLoc [44] to get the desired information for those proteins without subcellular location annotated in UniProt database. The updated website address for Euk-mPLoc can be found in the Cell-PLoc package [41] as well as in Table 3 of [45]. The good thing about Euk-mPLoc is that it not only can cover up to 22 subcellular location sites but is also able to identify proteins with multiple location sites, which is particularly useful for drug development as elaborated recently by Smith [46].

### KEGG enrichment scores of proteins

The simplest and most direct method for predicting the function of a query protein based on the training dataset of function-known proteins is the immediate neighborhood method [47]. The information of the neighbor proteins is also an important environmental feature to the protein concerned. Actually, the neighbor proteins are in interaction with each other in the STRING network [48]. The KEGG enrichment score of the protein and its neighbors was defined as the −log_10_ of the p value generated by hypergeometric test on KEGG pathway. The larger enrichment score means more overrepresentation. There were 220 KEGG enrichment scores for each of the proteins investigated here.

### Number of protein complexes

If a protein can form a complex with other proteins, it will be more stable and have longer half-life. Therefore, the number of this kind of complexes a protein can form is a feature relevant to its stability, and should be counted in prediction as well. We downloaded the protein complex dataset from CORUM [49], which is a comprehensive resource of mammalian protein complexes.

### Feature space of proteins

As mentioned above, the 7 types of biochemical and physicochemical properties would contribute 132 components to describe a protein. In addition, its length could also be counted as a component, its occurrences in the 22 subcellular location sites as 22 components, its 220 KEGG enrichment scores as 220 components, and its number in forming protein-protein complexes as a component, the total components used in this study to represent a protein sample would be (132+1+22+220+1) = 376 components. For the list of the 376 feature components, see the Table S1.

Thus, the 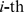 protein sample 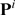 should be formulated as a vector in a 376-D (dimensional) space; i.e.,
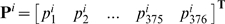
(1)where 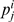 is the 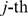 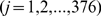 component of the 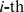 protein sample 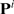 and can be derived by following the procedures as elaborated above.

Note that before performing prediction, each of the 376 components in Eq.1 should undergo the following standard conversion procedure:
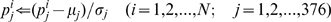
(1a)where 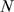 is the number of the total proteins in the training dataset, 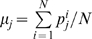 and 
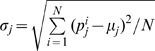
 are the mean and standard deviation of the 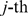 component over the 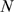 protein samples. The converted values obtained by Eq.1a will have a zero mean value over the 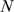 protein samples, and will remain unchanged if going through the same conversion procedure again [24], [26].

### mRMR method

The “maximum relevance & minimum redundancy” (mRMR) method was originally developed by Peng et al. [50] to deal with the microarray data processing. In their method, each feature is ranked according to its relevance to the target and redundancy with other features. A “good” feature is defined as the one that has the best trade-off between maximizing the relevance to the target and minimizing the redundancy within the features. To quantify both the relevance and redundancy, the following mutual information (MI) is defined to estimate how one vector is related to another:
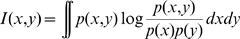
(2)where 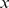, 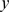 are two vectors, 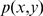 is the joint probabilistic density, 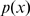 and 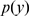 are the marginal probabilistic densities.

Suppose 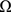 denotes the entire space containing all the aforementioned 376 components, and 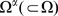 denotes the space contains 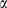 components selected from 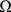. The space to be identified is denoted by 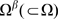 that contains 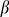 components. The relevance 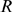 of the feature 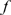 in 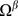 with the target 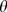 can be calculated by:
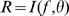
(3)And the redundancy 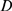 of the feature 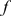 in 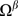 with all the features in 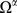 can be calculated by:
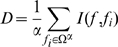
(4)To obtain a feature 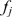 in 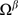 with maximum relevance and minimum redundancy, Eqs.3 and 4 are combined with the mRMR function:
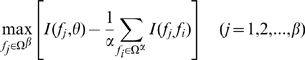
(5)


For a feature set with 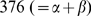 components, the feature evaluation will continue for 376 rounds. After these evaluations, a feature set 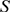 can be obtained by the mRMR method as formulated below:
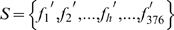
(6)where each feature in 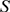 has an subscript index, indicating at which round that the feature is selected. The better a feature is, the earlier it will satisfy Eq.5 and be selected, and the smaller its subscript index will be.

### Nearest Neighbor Algorithm

In our study, the Nearest Neighbor (NN) algorithm or NNA is used to classify a protein as either labile or a stable one. NNA makes its decision by calculating the “distances” of a query protein with all the proteins in the training dataset one-by-one. Varieties of distance scales can be used for this purpose, such as Euclidean distance [51], Hamming distance [52], and Mahalanobis distance [34]. In the current study, the distance between the query protein 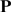 and 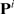, the 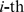 protein in the training dataset, is defined by [53], [54], [55]:
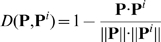
(7)Where 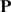 and 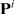 are the feature component vector of query protein and the 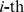 protein in the training dataset (cf. Eq.1); 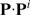 is the inner product of 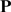 and 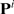; 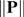 and 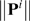 represent the modules of vectors 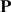 and 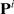. The smaller 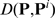 is, the more similar 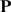 to 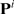 is. According to the NN rule, given a training set 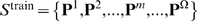, the query protein 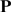 will be predicted belonging to the same class of 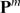 that is the closest to 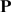. In other words, if
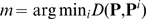
(8)where 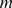 is the argument of 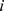 that minimizes 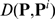, and if 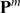 belongs to 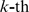 class, then the query protein 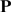 should also belong to the same class.

### Jackknife Cross-Validation Method

In biological literatures, the independent dataset test, subsampling or K-fold (such as 5-fold and 10-fold) test, and jackknife test are the three cross-validation methods often used to examine the accuracy of a statistical predictor [52]. Of these three, however, the jackknife is thought the most objective as elucidated in [41] and elaborated in [40]. Therefore, the jackknife cross-validation has been increasingly adopted to examine the power of various predictors (see, e.g., [54], [56], [57], [58], [59], [60]) and will be used in this study as well. During jackknifing, each protein sample in the benchmark dataset is in turn singled out to test using the rule parameters trained by the remaining protein samples. For clarity to describe the test process, let us define
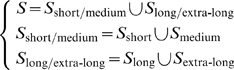
(9)where 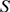 is the benchmark dataset used in this study (cf. Dataset S1), 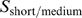 the sub-dataset containing only the “short” or medium” half-life proteins, 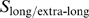 only the “long” and “extra-long” half-life proteins, 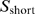 only the “short” half-life proteins, 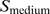 only the “medium” half-life proteins, 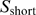 only the “long” half-life proteins, 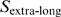 only the “extra-long” half-life proteins, and 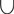 the union symbol in the set theory. The jackknife success rates were examined according to the following equations:
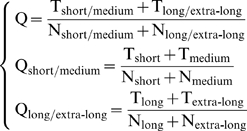
(10)where 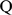 is the overall success rate in identifying proteins in 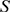 as “short/medium” or “long/extra-long” type (see the 1^st^ equation of Eq.9), 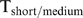 the number of corrected predictions for the “short/medium” type, 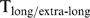 the number of corrected predictions for the “long/extra-long” type, 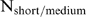 the number of total proteins in 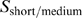, and 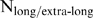 the number of total proteins in 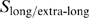; 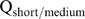 the success rate in identifying proteins in 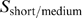 as “short” or “medium” type (see the 2^nd^ equation of Eq.9); 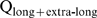 the success rate in identifying proteins in 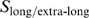 as “long” or “extra-long” type (see the 3^rd^ equation of Eq.9).

### Feature Selection

Although the mRMR step could arrange the feature components according to some sort of ranks, it is not sufficient for us to determine which feature components should be selected to optimize the performance of our predictor. To solve the problem, the IFS (incremental feature selection) method is adopted as illustrated below.

Based on the ranked features obtained from the mRMR step, we can construct 376 feature component sets by adding one component at a time in an ascending order, with the *i*-th set given by
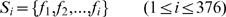
(11)For each of such 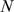 feature component sets, an NNA predictor was constructed and its jackknife success rate derived. Finally, we obtained a curve, called the IFS curve, with the subscript index *i* in Eq.11 as its *X*-axis and the corresponding jackknife success rate as its *Y*-axis. The feature set, say 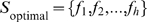, would be deemed as the optimal one if the IFS curve has a peak at 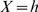.

### Predict metabolic stability of drug target proteins

We predicted the stability of 170 proteins targeted by 332 drugs with known half-life. The drug-target pairs and half-life of drugs were downloaded from DrugBank [61]. Only the drugs with well-defined target proteins and half-life were analyzed. To unify the time unit, the half-life spans of all the drugs investigated were uniformly converted to minutes. As formulated in Eqs.8 and 9, the test procedures are as follows. A query drug target protein was first identified as “short/medium” half-life and “long/extra-long” half-life. If it turned out to “short/medium” half-life, the predictor would automatically continue to classify it as “short” half-life or “medium” half-life; otherwise, classify it as “long” half-life or “extra-long” half-life. Finally, each of the drug target proteins investigated was assigned as “short”, “medium”, “long”, or “extra-long” half-life, respectively.

## Results

### mRMR results

The mRMR program in this study was downloaded from http://penglab.janelia.org/proj/mRMR/. We set the parameter 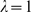 to characterize our data into three groups according to their values which are: (1) smaller than 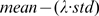, (2) between 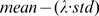 and 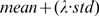, and (3) greater than 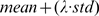. In the above criteria, 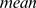 is the average value of the features in all samples, and 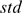 the standard deviation. In addition to the list generated by the mRMR to show the index of each feature described above, mRMR also output a table called MaxRel list that contains the relevance of features to their target, as defined in Eq.3. In this study, only the mRMR list was used in the feature selection procedure.

### IFS results

**Figure 2:**
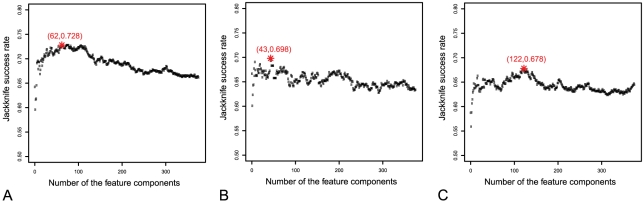
The IFS curves of protein's metabolic stability predictions. The IFS curves for (A) all the proteins in *S* (cf. Eq. 9), (B) only the “short” and “medium” half-life proteins in *S*_short/medium_, and (C) only the “long” and “extra-long” half-life proteins in *S*_long/extra-long_. The overall accuracy reached its peak of 72.8% when the number of feature components used for the classifier between “short/medium” half-life and “long/extra-long” half-life was 62. The corresponding accuracy peak and featured component number for the case of panel B are 69.8% and 43, while those for the case of panel C are 67.8% and 122.

In the IFS procedure, we built 376 feature sets based on the ordered feature set *S* obtained in the mRMR step. Accordingly, 376 prediction models were constructed and tested as described above. Shown in **Figure 2** is the IFS curve for (A) all the proteins in 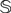 (cf. the 1^st^ equation of Eq.9), (B) only the “short” and “medium” half-life proteins (cf. the 2^nd^ equation of Eq.9), (C) only the “long” and “extra-long” half-life proteins (cf. the 3^rd^ equation of Eq.9). As shown in **Figure 2** (A), the overall accuracy reached its peak of 72.8% when the number of feature component used was 62. The 62 feature components selected by mRMR would constitute the optimal feature set for the “short/medium”-“long/extra-long” classifier. The optimal feature set for the “short”-medium” classifier contained 43 feature components, with the peak success rate of 69.8%; while the optimal feature set for the “long”-“extra-long” classifier contained 122 feature components, with the peak success rate of 67.8%. The optimal feature components were extracted according to their impact to the success rates in predicting stability of proteins. The aforementioned 62, 43, and 122 optimal feature components are provided in the Table S2 (A), (B), and (C), respectively.

### Analysis of optimal feature components

**Figure 3:**
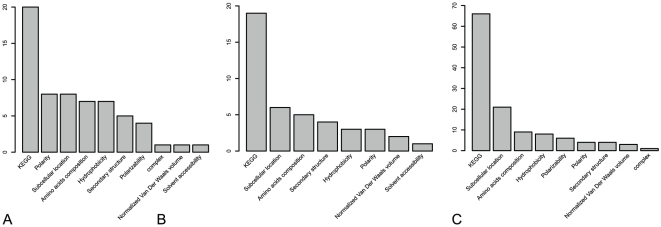
The numbers of each kind of features in optimal feature sets. The numbers of each kind of features for (A) the 62 feature components in the optimal “short/medium”-“long/extra-long” classifier, (B) the 43 feature components in the optimal “short”-“medium” classifier, and (C) the 122 feature components in the optimal “long”-“extra-long” classifier.

To investigate what kinds of features are critical for protein stability, we extracted the optimal feature components and counted the numbers of each kind of features. Shown in **Figure 3** is the numbers of each kind of features in (A) the 62 feature components for the “short/medium”-“long/extra-long” classifier, (B) the 43 feature components for the “short”-“medium” classifier, and (C) the 122 feature components for the “long”-“extra-long” classifier, respectively. As we can see from **Figure 3**, the following seven kinds of features play the major roles in affecting the protein stability: (1) KEGG enrichment scores, (2) subcellular locations, (3) polarity, (4) amino acids composition, (5) hydrophobicity, (6) secondary structure propensity, and (7) the number of protein complexes.

In a recent work Yen et al. [23] discovered that protein stability was correlated with amino acid composition. Our results have further confirmed their finding. These authors also found that the short half-life group and medium half-life group had a larger proportion of the unstable “cell cycle control” proteins, and that the long half-life group had a larger fraction of “mitosis” proteins consisting of actins, tubulins, septins, and so forth. Interestingly, our studies indicate that the metabolic stability of a protein is associated with its subcellular location, such as whether it is located in nucleus, cytoplasm, extracellular, or cell membrane, quite consistent with their findings [23] as well. Meanwhile, it was found that the enrichment of degradation, metabolism and signaling pathways could help predict protein's metabolic stability (see Table S2), which is quite sensible as well.

Proteins bound with ligands or proteins not prone to be denatured, are usually more stable. This would logically require them to have proper fold patterns or microenvironments. The reason why membrane proteins are relatively more stable is that their folding process involves binding with, transmembrane helix insertion into (see, e.g., [3], [62], [63]), and helix-helix interactions with the presence of bilayer interfaces [64], [65], [66]. Membrane protein fold topology may be categorized into two basic secondary structural motifs, namely α-helices and β-barrels [67]. Stability is a consequence of the low electrostatic potential energy of small substructures called knots and is opposed by the stress developed in contraction of the large substructures called matrices [68]. The features investigated in this study have provided useful insight regarding the energetics of driving forces governing folding, assembly, insertion, and translocation of membrane proteins [69]. The knowledge of inter-residue interactions in proteins structures is very useful for understanding the mechanism of protein folding and stability. Also, the secondary structure propensity of amino acids in a protein, as well as their polarity and hydrophobicity, would play an important role to the inter-residue interactions, and hence to its fold pattern [70], folding rate [71], [72], and stability as well [73]. Furthermore, driven by the hydrophobic force, a protein could overcome the entropic barrier and fold from a random coiled state into some type of topological shape, with disulfide bonding, hydrogen bonding, ion-pairs, and van der Waals interactions defining the shape and keeping it from falling apart [74].

A general solution for predicting the metabolic stability of proteins, even with a moderate success rate, is an extremely difficult and complicated problem. However, any progress in this regard would provide us with very useful insights for in-depth researches in protein science and developing new strategy for drug design.

### The predicted metabolic stability of drug target proteins

It is interesting to predict the metabolic stability of drug target proteins and compare the results with the half-life spans of the corresponding drugs. Although there were many factors that can affect the half-life of a drug, we found that the stability of its target protein is a quite important one. For demonstration, the predicted metabolic stability outcomes for some drug target proteins and the real half-life spans of their corresponding drugs are given in the Table S3, from which we found some intriguing correlations. The half-life of drugs targeted to proteins with predicted “short or medium half-life” (with median of 420 minutes) was shorter than the half-life of drugs targeted to proteins with predicted “long or extra-long half-life” (with median of 709 minutes). The median of the half-life of drugs targeted to proteins with predicted “short half-life”, “medium half-life”, “long half-life” and “extra-long half-life” were 303, 510, 540 and 1080 minutes, respectively.

For instance, Dinoprostone (DrugBank accession number DB00917) is a prescription drug used, as a vaginal suppository, to prepare the cervix for labour and to induce labour. The half-life of Dinoprostone is less than 5 minutes. The predicted stability results for its target proteins PTGER1 (UniProtKB/Swiss-Prot ID P34995) and PTGER2 (UniProtKB/Swiss-Prot ID P43116) were both “short” half-life. Again, Clorazepate (DrugBank accession number DB00628) is for treating anxiety. It also has the function for muscle relaxant and anticonvulsant. Its half-life is about 2 days (1,440 minutes), and the predicted stability for its target proteins BZRP (UniProtKB/Swiss-Prot ID P30536) and GABRA1 (UniProtKB/Swiss-Prot ID P14867) were “long” and “extra-long”, respectively, fully consistent with the sense that the more stable a protein is, the longer half-life drug is needed for effectively targeting it; and vice versa.

## Discussion

We have developed a new method for predicting the metabolic stability of proteins by integrating their various biochemical and physicochemical features. It is indicated by the rigorous jackknife cross-validation test that the predictor can achieve an overall success rate of 72.8%. With the feature selection approach based on the mRMR method and IFS procedure, we found that the following seven features would play the major roles in determining the stability of proteins: KEGG enrichment scores, subcellular locations, polarity, amino acids composition, hydrophobicity, secondary structure propensity, and the number of protein complexes. These findings might provide useful information for drug development. The method presented in this paper might also become a high throughput tool for large-scale annotating the metabolic stability of proteins.

## Supporting Information

Dataset S1 The sequences of benchmark dataset.
(0.71 MB TXT)

Table S1 List of the 376 feature components.
(0.10 MB XLS)

Table S2 The optimal feature components.
(0.05 MB XLS)

Table S3 The predicted metabolic stability for drug target proteins.
(0.10 MB XLS)
